# Supplementary material for: Interleukin-1 Receptor Antagonist Modulates Liver Inflammation and Fibrosis in Mice in a Model-Dependent Manner
Source: Int J Mol Sci. 2019 Mar 14;20(6):1295. doi: 10.3390/ijms20061295 (PMC6471711; doi:10.3390/ijms20061295)
Supplement: Supplementary file 1 [file ijms-20-01295-s001.pdf]

International Journal of *Molecular Sciences*

MDPI

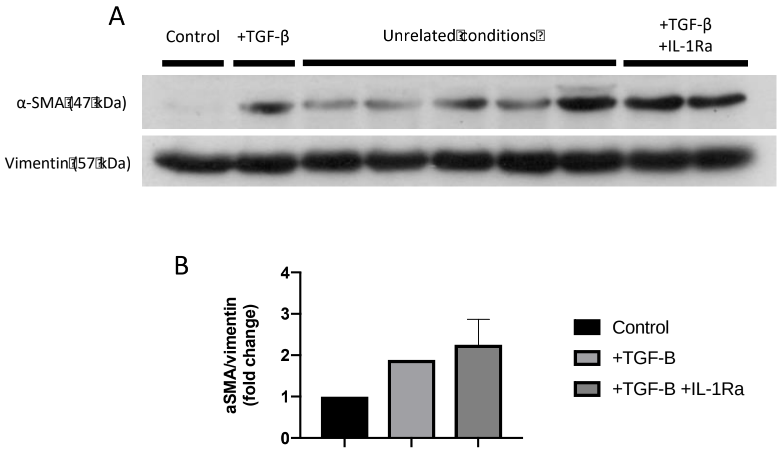

Supplementary Figure 1. IL-1Ra does not reverse TGF-β1-dependant stellate cell activation. (A) Total protein extracts from untreated LX-2 human hepatic stellate cells (LX-2) (control), LX-2 treated with TGF-β, and LX-2 treated with TGF-β and IL-1Ra simultaneously were obtained. Protein extracts were subjected to SDS-PAGE, transferred to nitrocellulose and blotted with anti-α-SMA and vimentin. (B) α-SMA signals were quantified by densitometry and normalized using vimentin signals as a loading control.

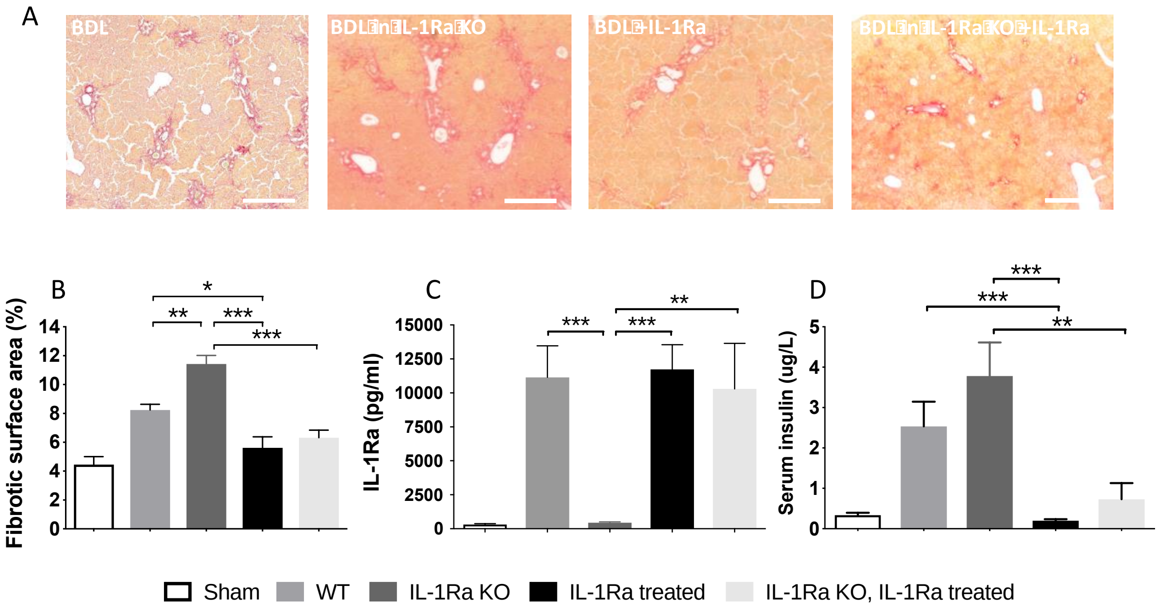

Supplementary Figure 2. IL-1Ra knockout mice rescue with IL-1Ra treatment. Mice livers were fixed in formalin and embedded in paraffin following 2-4 weeks BDL. Groups presented in figure 3 were compared to IL-1Ra KO mice treated with IL-1Ra treated mice (50 mg/kg/day) (n=5). Liver sections

stained by sirius red (A). On sirius staining liver parenchyma appears in light yellow and fibrotic areas in red. Morphometric quantification of fibrosis was performed on multiple liver sections and expressed as percentage of liver surface area (B). Serum levels of IL-1Ra (C) and fasting insulin (D) were measured by ELISA. Scale bars, 400 μm. * $p < 0.05$, ** $p < 0.01$, *** $p < 0.001$, comparing two groups as indicated.

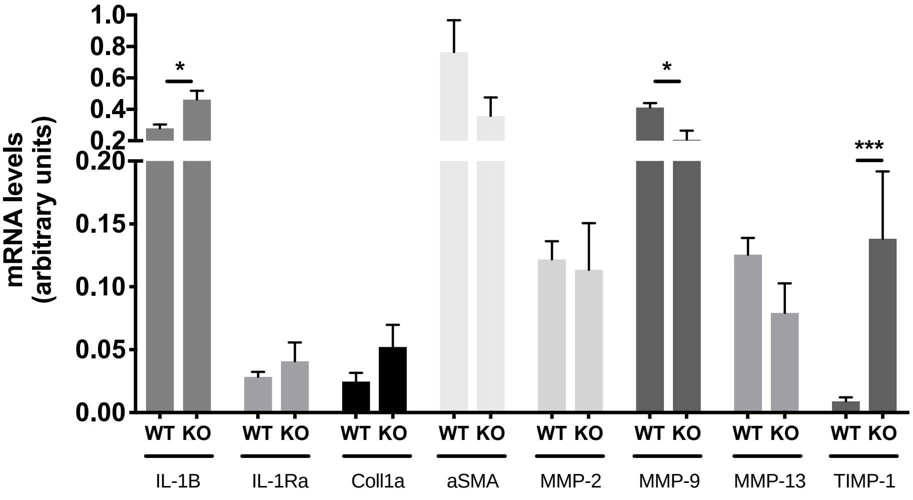

Supplementary Figure 3. Comparison between wild type (WT) and knockout (KO) baseline mRNA levels of fibrosis-related gene of interest. IL-1β, IL-1Ra, collagen type I, α-SMA, matrix metalloproteinase (MMP) 2, 9 and 13, tissue inhibitor of metalloproteinase 1 (TIMP-1) baseline mRNA levels in non-fibrotic sham wild type and IL-1Ra KO mice are represented (3-8 animals per group). * $p < 0.05$, ** $p < 0.01$, *** $p < 0.001$, comparing two groups as indicated.
